# Supplementary material for: Mapping structure heterogeneities and visualizing moisture degradation of perovskite films with nano-focus WAXS
Source: Nat Commun. 2022 Nov 5;13:6701. doi: 10.1038/s41467-022-34426-y (PMC9637205; doi:10.1038/s41467-022-34426-y)
Supplement: Supplementary file 1 — Supplementary Information [file 41467_2022_34426_MOESM1_ESM.pdf]

Supplementary information

Mapping structure heterogeneities and visualizing moisture degradation of perovskite films with nano-focus WAXS

Nian Li[1‡], Shambhavi Pratap[1‡], Volker Körstgens[1], Sundeep Vema[2,3], Lin Song[4], Suzhe Liang[1], Anton Davydok[5], Christina Krywka[5] and Peter Müller-Buschbaum[1,6*]

Supplementary Note 1. Williamson-Hall method[1,2]

Williamson and Hall proposed an approximation, where the integral breadth β consists of size-induced and strain-induced broadening. The crystallite size contribution β_D is analyzed by the Scherrer equation

$$\beta_D = \frac{K\lambda}{Dcos\theta} \quad (1)$$

The shift of each peak ($\Delta\theta$) caused by the strain ($\varepsilon = \Delta d/d$) is calculated by differentiating Bragg's law.

$$\Delta\theta = -\varepsilon\, tan\theta \quad (2)$$

Via combining the above equations, the general expression for the integral breadth β is given by Williamson and Hall.

$$\beta cos\theta = 4\varepsilon sin\theta + \frac{K\lambda}{D} \quad (3)$$

This allows a linear fit to be realized. The slope/4 reflects the microstrain change, and the y-intercept indicates the crystallite size contribution.

Supplementary Note 2. Estimation of the strain variation

The q postions are extracted via fitting Gaussian functions for the (100), (110) and (111) Bragg peaks of the perovskite films (Supplementary Figure 14) as shown in Supplementary Figure 15. As reported by Jones et al.[2], microstrain dominates the peak breadth, and thus the residual strain is estimated using the relative shift of the peak q-value from the reference value. Here, we use $(q_{max} - q)/q_{max}$ to estimate the strain variation. The positions of the Bragg peaks of the perovskite film at the depth of 20 μm is taken for q_{max}, as a reference value (without strain).

Supplementary Note 3. Factors related to stress (strain)

The correlation between stress (σ) and thermal expansion mismatch is quantified by[3]:

$$\sigma_{\Delta T} = \frac{E_P}{1-v_P}(\alpha_S - \alpha_P)\Delta T \quad (4)$$

E_P is the modulus of the perovskite, v_P is Poisson's ratio in the perovskite, α_S and α_P are the thermal expansion coefficients of the substrate and the perovskite, respectively. The factors can be mainly divided into two categories: (1) the temperature gradient during cooling from the annealing temperature of the perovskite film to room temperature ΔT; (2) the difference in thermal expansion coefficients (α) between the perovskite and the contacting layers $\Delta\alpha$.

For the perovskite compound, v_P is larger than 0.3. The bulk, shear, Young's modulus ranges are within 12-30 GPa, 3-12 GPa, and 15-37 GPa, respectively. Furthermore, Young's modulus at different crystallographic planes exhibit strong anisotropic properties for all $CH_3NH_3BX_3$ (B = Sn, Pb; X = Br, I)[4].

The above factors generally influence strain across the whole film. Other factors like compositional material inhomogeneity, phase transition, grain boundary, etc, primarily affect the local strain[5].

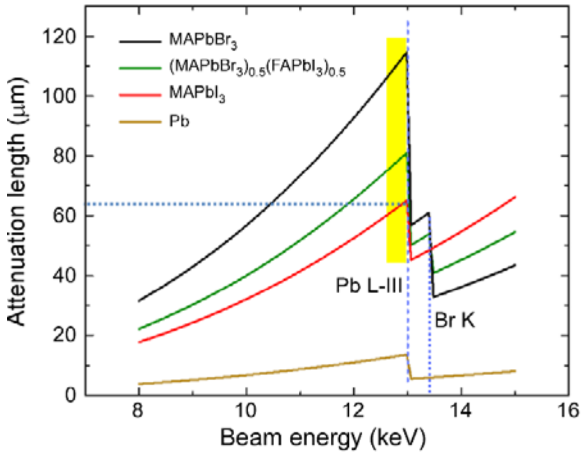

Supplementary Figure 1. Attenuation length of the X-ray beam for the element Pb and perovskite compounds. The absorption energy of the Pb L-III edge is located at around 13.04 keV, and the absorption energy of the Br K edge is located at around 13.47 keV. Thus, the X-ray beam energy was chosen at 12.75 keV to minimize beam damage for the investigated film, and well suited for high-transmission signals.

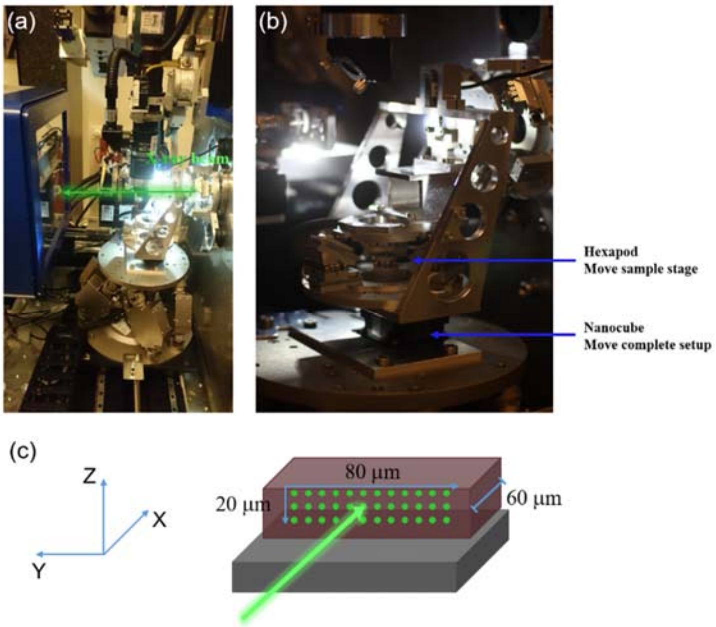

Supplementary Figure 2. Synchrotron radiation-based nWAXS measurement setup. (a)(b) Photographs of the nWAXS experiment at the synchrotron beamline with the characteristic components indicated. (c) Schematic illustration of the nWAXS experiment: performing the matrix scan on the perovskite film. The perovskite film with the width of ~60 μm is positioned on the edge of a steel substrate to prevent shadowing of the lower hemisphere of the scattering pattern. The scanned area is 80 μm × 20 μm (length × depth).

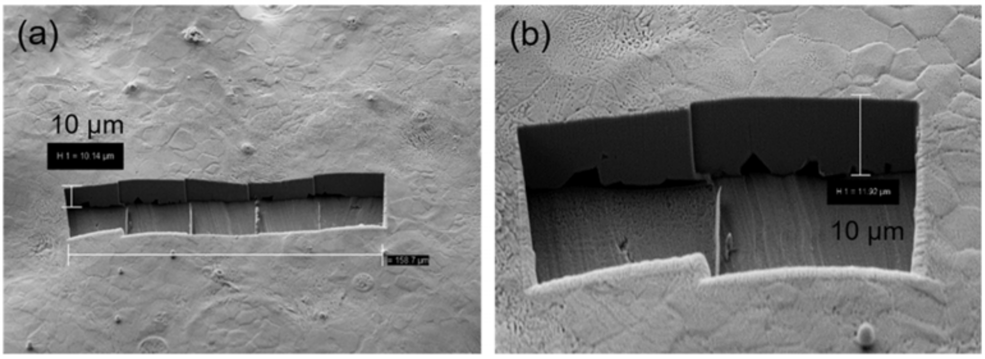

Supplementary Figure 3. Examples of FIB milling of the perovskite film. (a) Five and (b) two consecutive FIB milling areas showing clean etches. The sample is tilted with respect to the electron beam (around 54°) for the experiments.

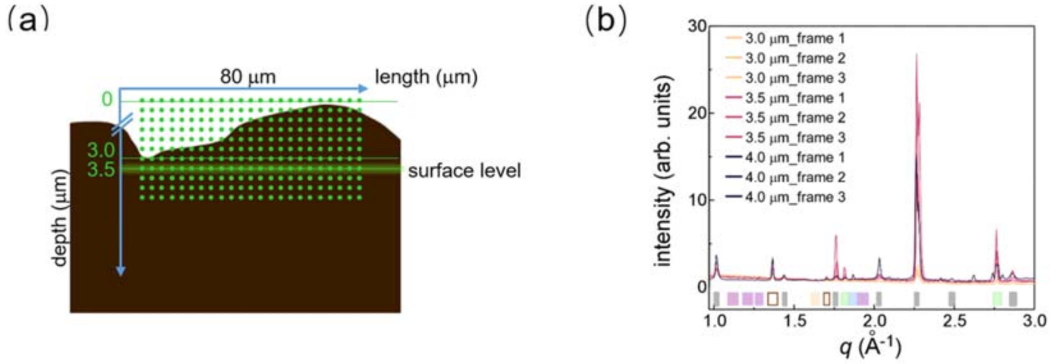

Supplementary Figure 4. Mapping dimension of 80 µm length × 20 µm depth and sample surface. (a) An illustration of the mapping dimension and sample surface. (b) A comparison of the first three frames at the depth of 3.0 µm, 3.5 µm and 4.0 µm. In (b), the scattering signal intensities of first three frames at the depth of 3.5 µm are higher than those at 3.0 µm, and comparable with those at 4.0 µm. Thus, we assume that due to the rough surface and the milling precision of the FIB, the nano X-ray beam directly travels through air or illuminates a small partial sample at the beginning of the scans (depth < 3.5 µm; details in Supplementary Figure 5). At a depth = 3.5 µm, the measured spots (160 frames) show a similar scattering signal intensity as deeper inside the film, so that we define it as the surface level.

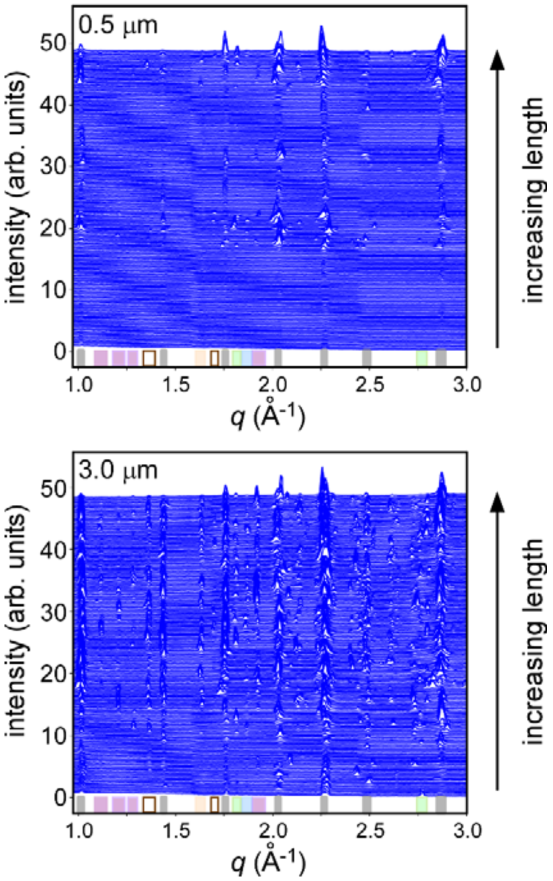

Supplementary Figure 5. Radially integrated line profiles of 2D nWAXS data of the $(MAPbBr_3)_{0.50}(FAPbI_3)_{0.50}$ film at the depth of 0.5 μm and 3.0 μm. Note that at the depth of 0.5 μm, no intensity peaks appear in the bottom profiles, indicating that the nano X-ray beam travels through air (as shown in Supplementary Figure 4), and thus signals originate from the background. With increasing depth, the nano X-ray beam gradually illuminates the sample or partial sample. In comparison, the profiles at the depth of 3.0 μm display numerous and clear intensity peaks. With increasing depth, each frame shows scattering signals from the sample (160 frames; 80 μm). The diffraction peaks are indexed and labeled with different colors (see the details in Supplementary Figure 10).

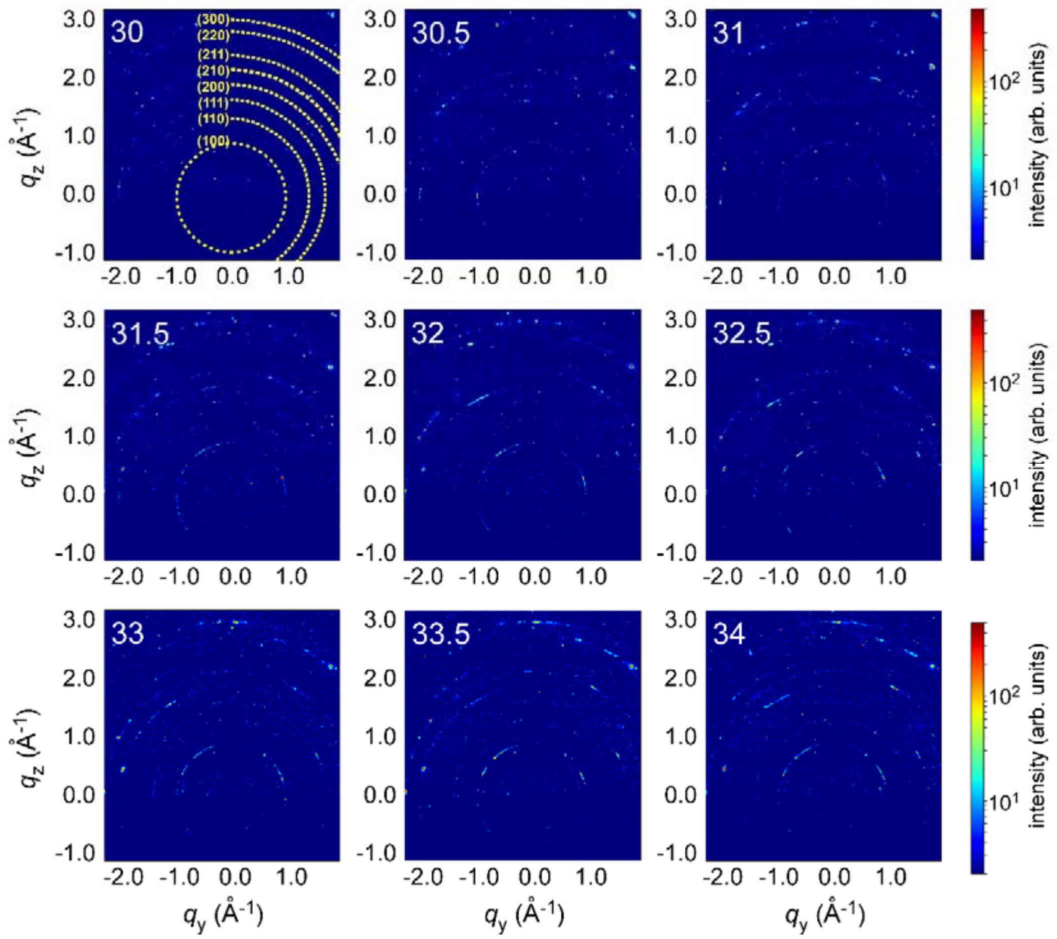

Supplementary Figure 6. Example of a series of neighboring individual 2D nWAXS images of the $(MAPbBr_3)_{0.50}(FAPbI_3)_{0.50}$ film. Selected 2D nWAXS images at the depth of 3.5 μm as a function of the sample length: 30 μm, 30.5 μm, 31 μm, 31.5 μm, 32 μm, 32.5 μm, 33 μm, 33.5 μm, and 34 μm. These images show low intensity of diffraction spots, due to the limited illuminated materials. The diffraction pattern rings, which correspond to a cubic perovskite phase, are labeled.

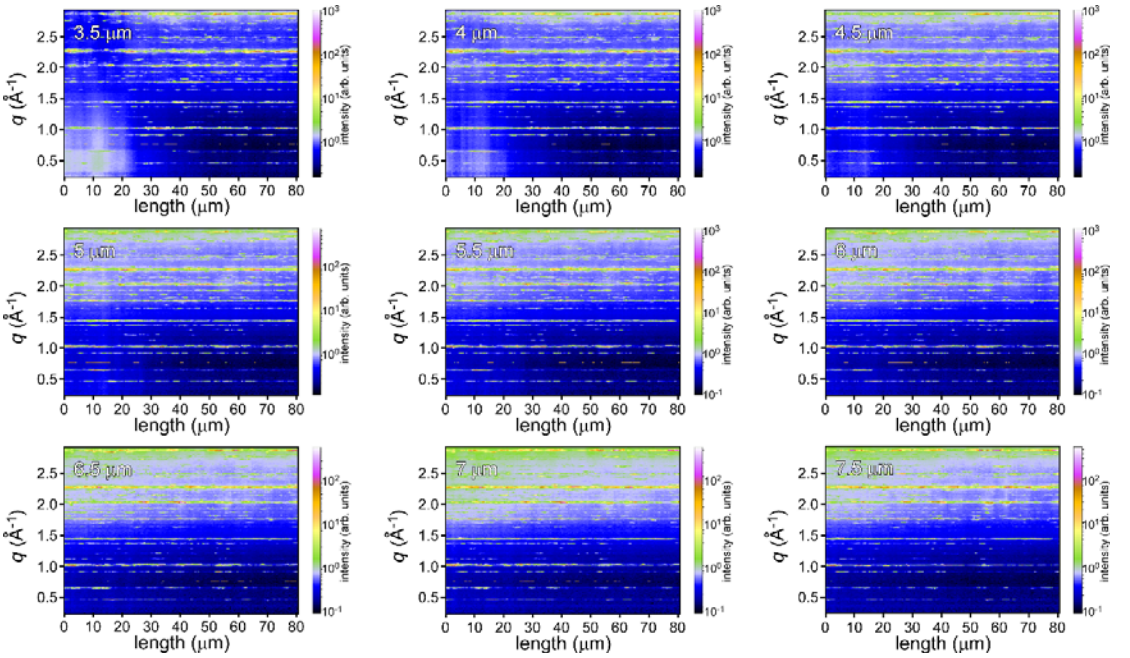

Supplementary Figure 7. Maps of radially integrated 2D nWAXS data of the top layer of the $(MAPbBr_3)_{0.50}(FAPbI_3)_{0.50}$ film. Selected 2D intensity mapping of *q* as a function of the sample length at different depths: 3.5 µm, 4 µm, 4.5 µm, 5 µm, 5.5 µm, 6 µm, 6.5 µm, 7 µm, and 7.5 µm.

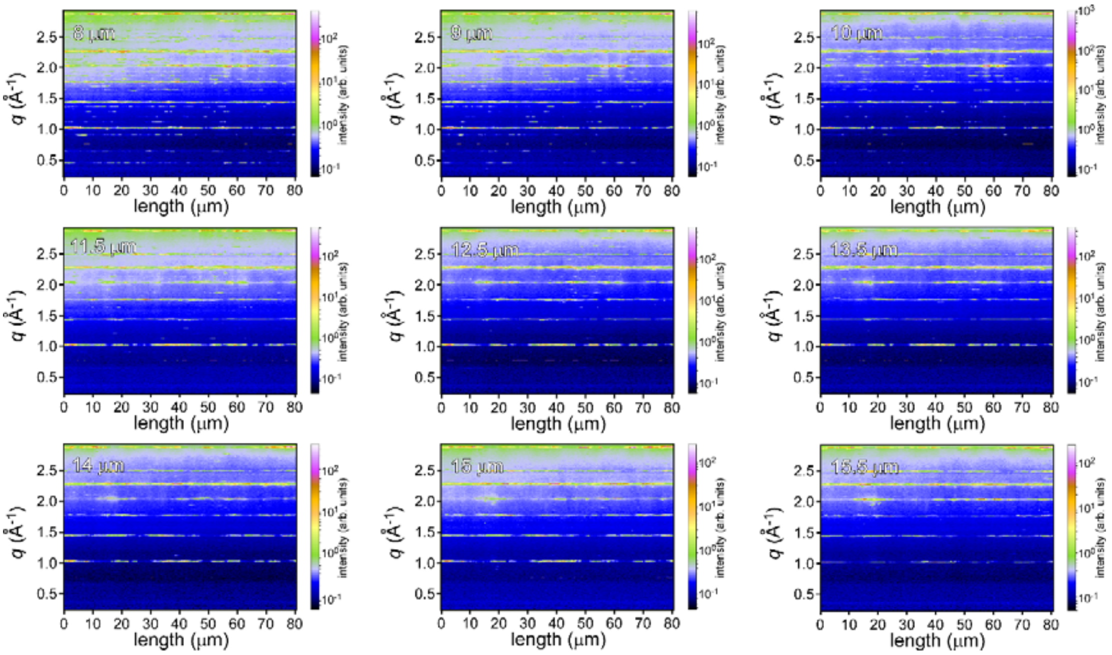

Supplementary Figure 8. Maps of radially integrated 2D nWAXS data of the middle layer of the $(MAPbBr_3)_{0.50}(FAPbI_3)_{0.50}$ film. Selected 2D intensity mapping of q as a function of the sample length at different depths: 8 µm, 9 µm, 10 µm, 11.5 µm, 12.5 µm, 13.5 µm, 14 µm, 15 µm, and 15.5 µm.

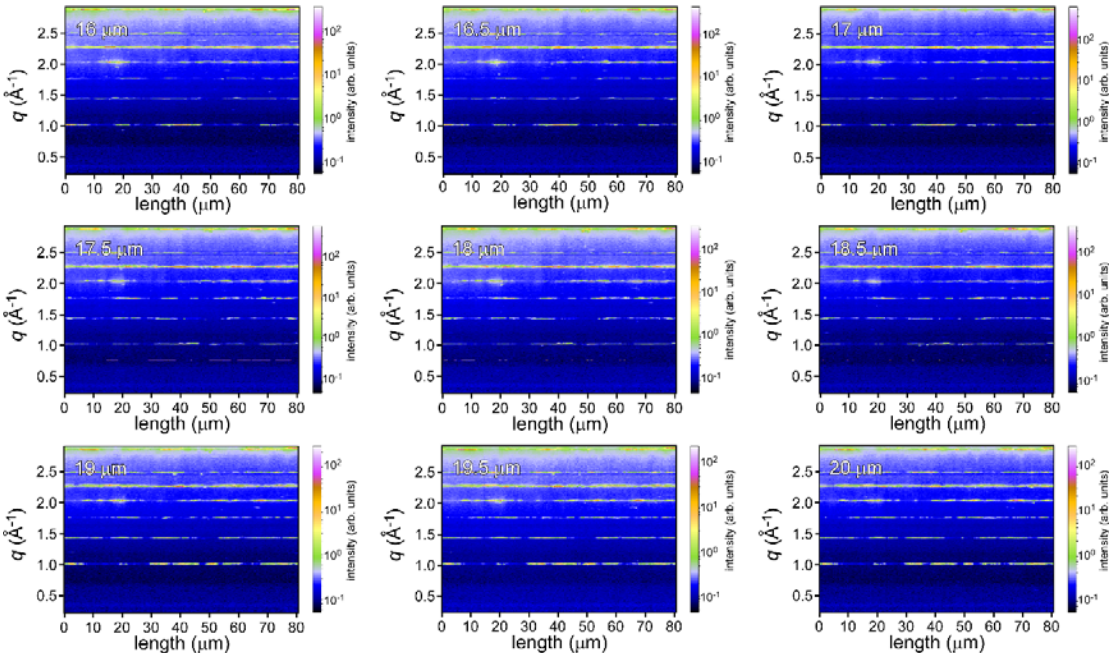

Supplementary Figure 9. Maps of radially integrated 2D nWAXS data of the bottom layer of the $(MAPbBr_3)_{0.50}(FAPbI_3)_{0.50}$ film. Selected 2D intensity mapping of *q* as a function of the sample length at different depths: 16 µm, 16.5 µm, 17 µm, 17.5 µm, 18 µm, 18.5 µm, 19 µm, 19.5 µm, and 20 µm.

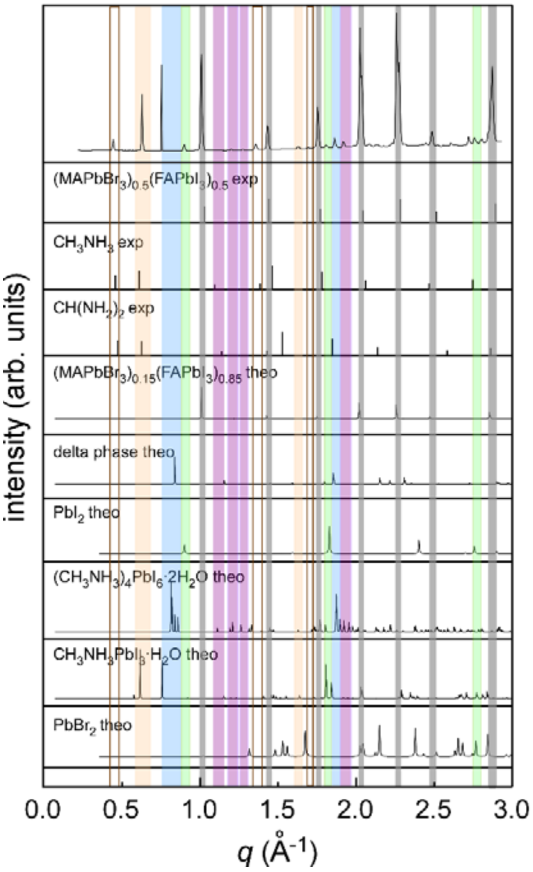

Supplementary Figure 10. Identification of the composition of the $(MAPbBr_3)_{0.50}(FAPbI_3)_{0.50}$ film. From top to bottom, the exemplary integrated line profile from the summed scattering data at the depth of 5 µm (160 frames), experimentally measured data[6,7] and simulated XRD patterns (CIF files; either from the published reference[8–12] or Crystallography Open Database/Cambridge Crystallographic Data Centre, see Supplementary Data 1) are compared, to enable a clear identification of the diffraction peaks as a cubic $(MAPbBr_3)_{0.50}(FAPbI_3)_{0.50}$ structure and the moisture-induced degradation products. The main index peaks of perovskite are indicated with black, monohydrate ($CH_3NH_3PbI_3 \cdot H_2O$) indicated with yellow, dihydrate ($(CH_3NH_3)_4PbI_6 \cdot 2H_2O$) indicated with purple, PbI_2 indicated with green, $CH_3NH_3Br/CH(NH_2)_2I$ indicated with bronze, and δ-phase indicated with blue. The colors are transparent for clarity of the presentation.

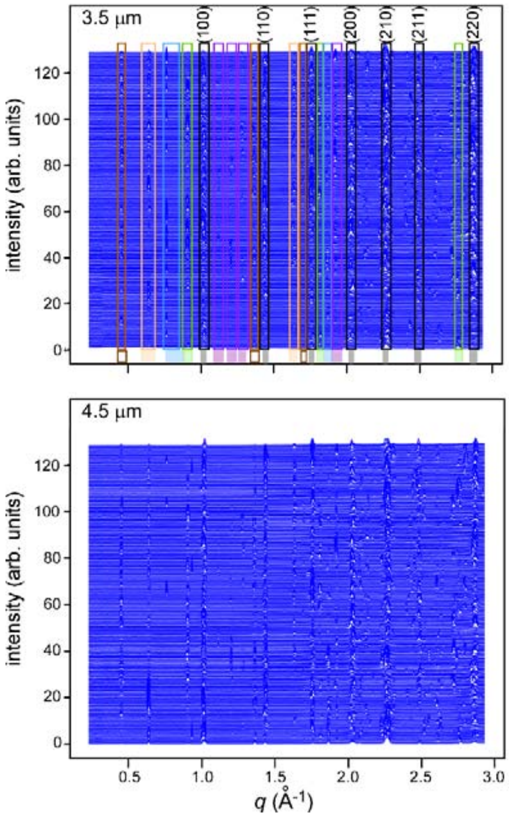

Supplementary Figure 11. Radially integrated line profiles of 2D nWAXS data of the top layer of the $(MAPbBr_3)_{0.50}(FAPbI_3)_{0.50}$ film. Selected radial integrations of 2D nWAXS data of the perovskite film as a function of the sample length at different depths: Examples of 3.5 μm, and 4.5 μm. The main index peaks of perovskite are indicated with black, monohydrate indicated with yellow, dihydrate indicated with purple, PbI_2 indicated with green, $CH_3NH_3Br/CH(NH_2)_2I$ indicated with bronze, and δ-phase indicated with blue[6,7,9,10,13]. The $PbBr_2$ crystal structure is not observed, might because the inclusion of Br contributes to a more stable structure resistant to the moisture degradation[14,15].

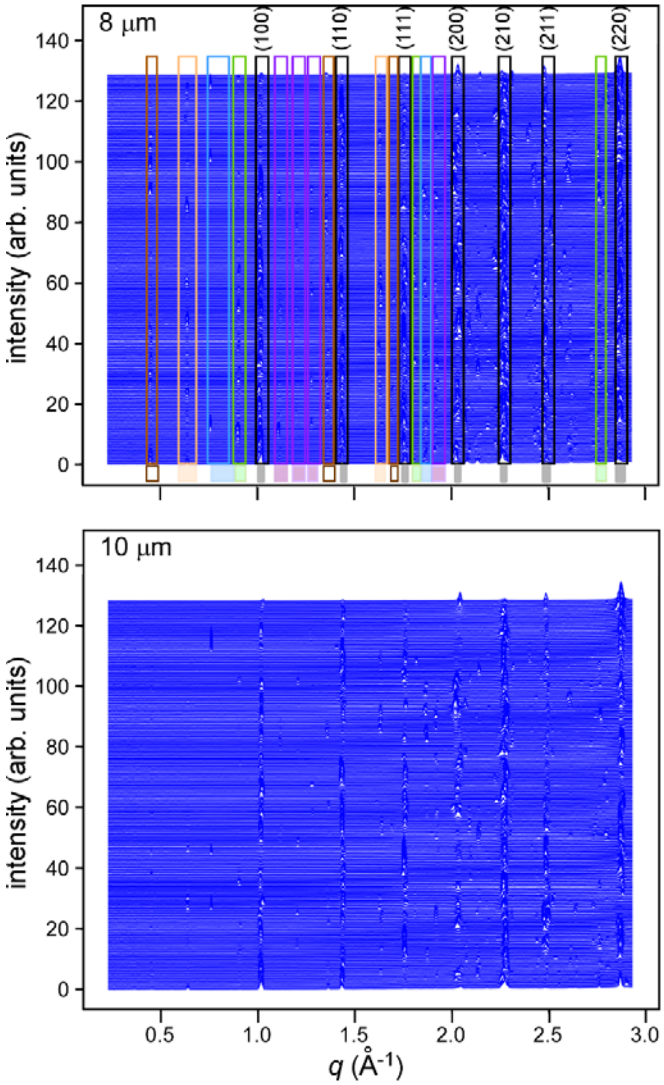

Supplementary Figure 12. Radially integrated line profiles of 2D nWAXS data of the middle layer of the $(MAPbBr_3)_{0.50}(FAPbI_3)_{0.50}$ film. Selected radial integrations of 2D nWAXS data of the perovskite film as a function of the sample length at different depths: Examples of 8 μm, and 10 μm. The main index peaks of perovskite are indicated with black, monohydrate indicated with yellow, dihydrate indicated with purple, PbI_2 indicated with green, $CH_3NH_3Br/CH(NH_2)_2I$ indicated with bronze, and δ-phase indicated with blue[6,7,9,10,13].

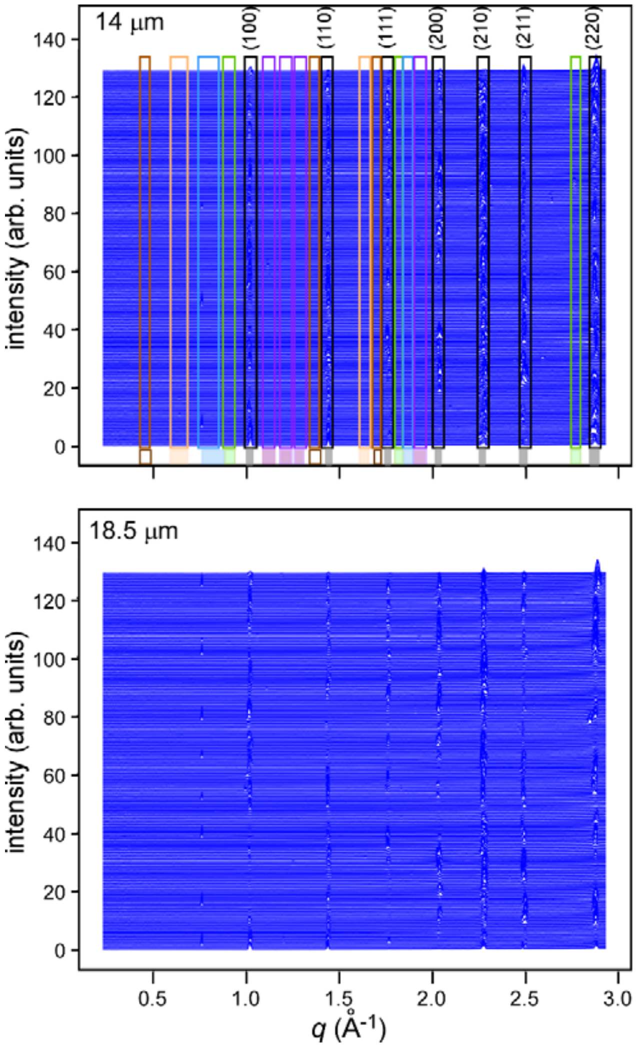

Supplementary Figure 13. Radially integrated line profiles of 2D nWAXS data of the bottom layer of the $(MAPbBr_3)_{0.50}(FAPbI_3)_{0.50}$ film. Selected radial integrations of 2D nWAXS data of the perovskite film as a function of the sample length at different depths: Examples of 14 μm, and 18.5 μm. The main index peaks of perovskite are indicated with black, monohydrate indicated with yellow, dihydrate indicated with purple, PbI_2 indicated with green, $CH_3NH_3Br/CH(NH_2)_2I$ indicated with bronze, and δ-phase indicated with blue[6,7,9,10,13].

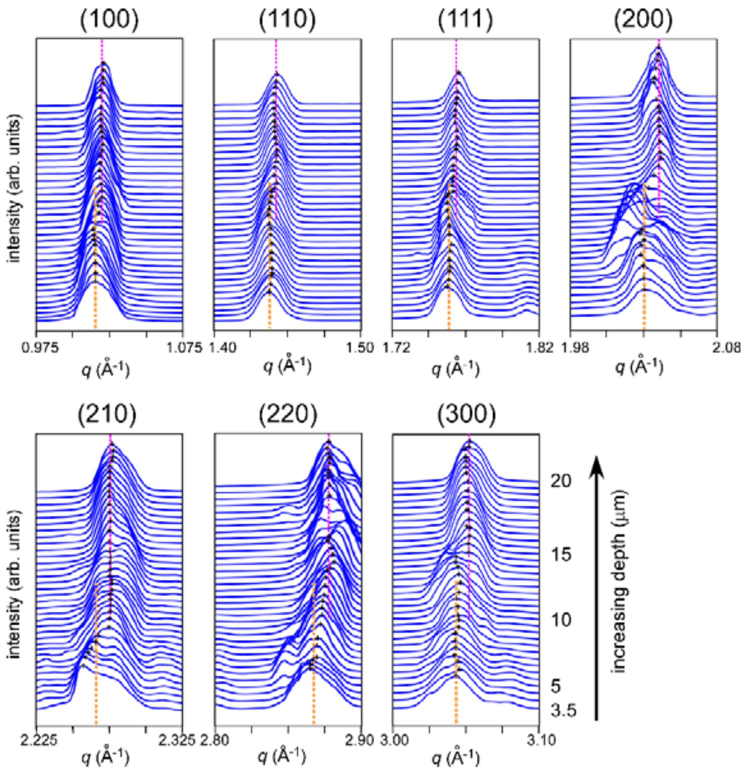

Supplementary Figure 14. Radially integrated line profiles of the summed 2D nWAXS data of the $(MAPbBr_3)_{0.50}(FAPbI_3)_{0.50}$ film (scanning a large length scale of 80 μm) as a function of the depth. In general, with the depth increase, the peak position shifts from the lower *q* value (indicated with the yellow dashed line) to the higher *q* value (indicated with the purple dashed line). Besides, we can also observe the peak broadening, which indicates the presence of the local complex structural inhomogeneity.

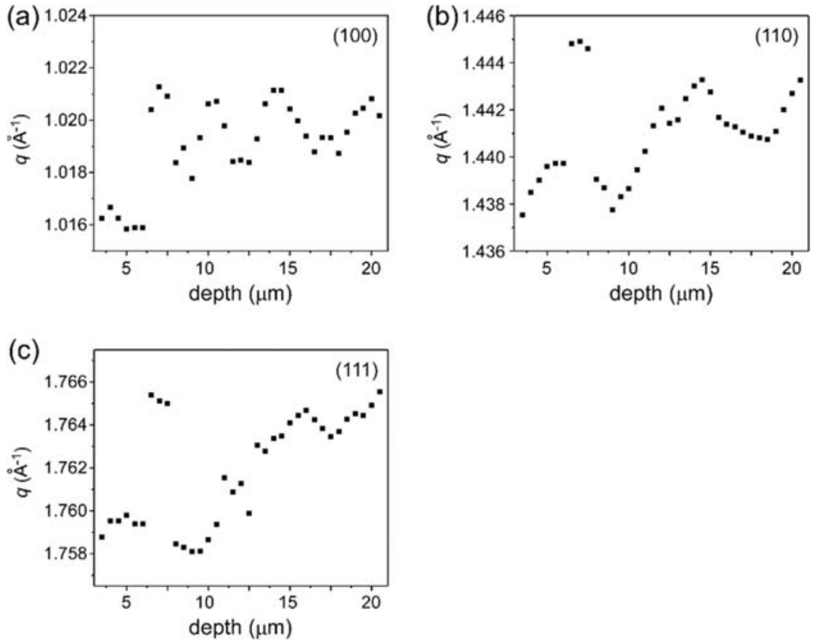

Supplementary Figure 15. Variations in Bragg peak *q* position as a function of depth. Radially integrated line profiles of the summed 2D nWAXS data of the $(MAPbBr_3)_{0.50}(FAPbI_3)_{0.50}$ film (scanning a large length scale of 80 μm) in Supplementary Figure 14 are fitted with a Gaussian function to extract the *q* positions of the (a) (100), (b) (110) and (c) (111) peaks. Overall, the (100), (110) and (111) *q* positions increase along with an increase in the depth.

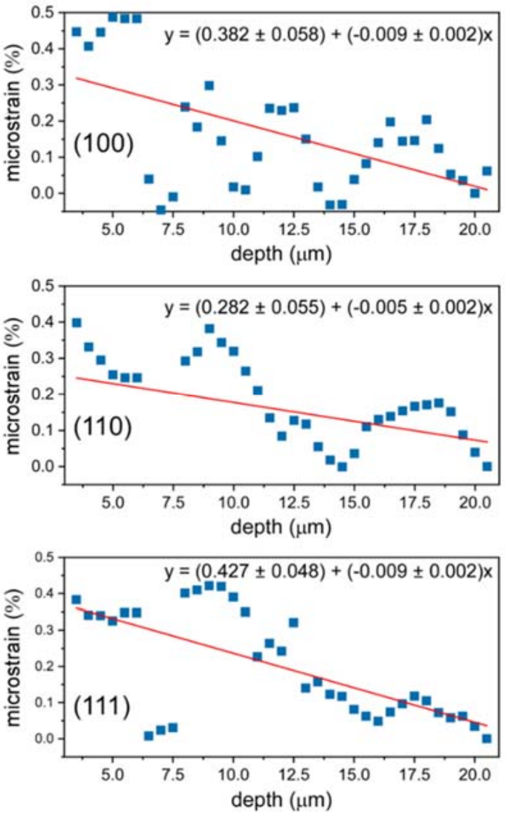

Supplementary Figure 16. Statistically-significant correlation of microstrain and depth. The microstrain is estimated via the Supplementary Note 2. A simple linear fit to the scattered data reveals a statistically-significant correlation (solid red line). Notably, to unveil the nature of the depth-dependent strain, a rigorous physical model would be required and probably a simpler perovskite system would be more suitable. Also with non-linear models, the statistically-significant correlation would not change the decreasing tendency of the microstrain versus depth.

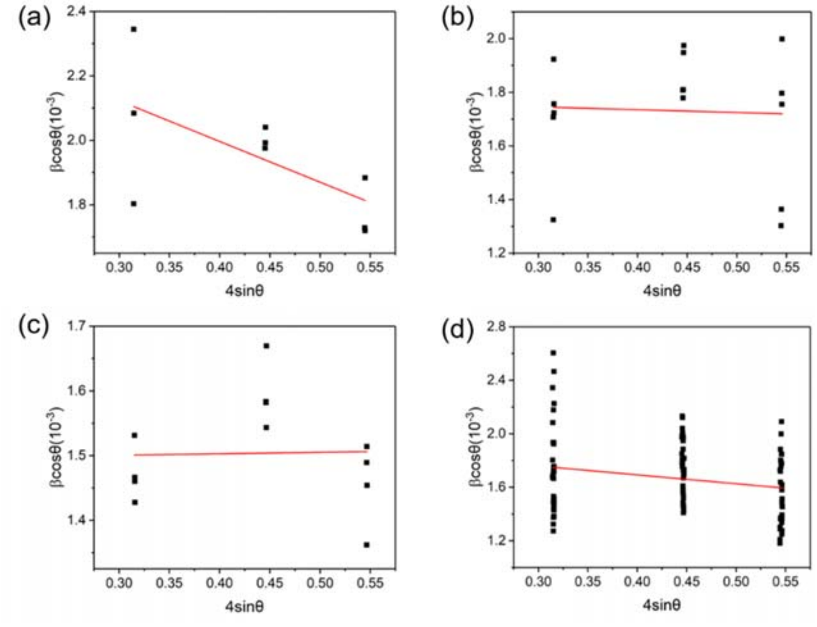

Supplementary Figure 17. Microstrain variations as function of depth using Williamson-Hall analysis. (a) Top region, (b) middle region, (c) bottom region and (d) whole film. In (a)-(d), the β, θ, and slope of the linear fit reflect the full width at half maximum, the Bragg peak and the microstrain. To obtain the microstrain change from the top region (a), to middle region (b), and then to bottom region (c), we use the radial integration profiles (Supplementary Figure 14) at the depth of 3.5-4.5 μm, 10-12 μm and 18.5-20 μm respectively for the calculation with the Williamson-Hall method (Supplementary Note 1). In (c), the slope is close to zero, indicating strain-free material in the bottom region. The microstrain result from the Williamson-Hall analysis is consistent with the estimation mentioned in the Supplementary Note 2 and Fig. 2b.

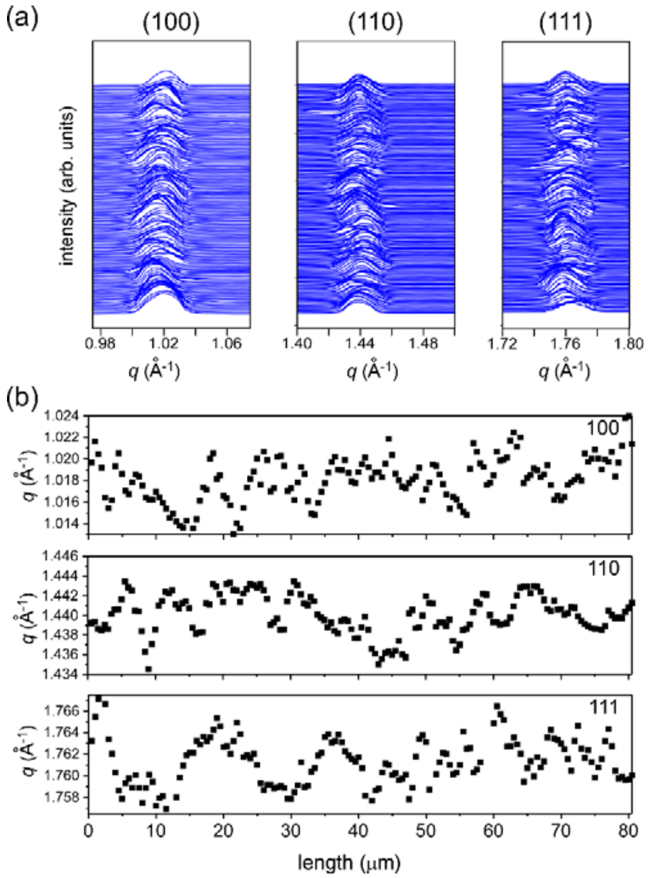

Supplementary Figure 18. Statistical analysis of *q* position variations in the horizontal direction. (a) Radially integrated line profiles of the vertical-summed 2D nWAXS data of the $(MAPbBr_3)_{0.50}(FAPbI_3)_{0.50}$ film (35 frames; a large depth of ~20 μm is summed), (b) variations in (100), (110) and (111) peak *q* position as a function of length. The *q* profiles in (a) are fitted with a Gaussian function to extract the *q* positions, shown in (b). Overall, no obvious tendencies are observed along the horizontal direction, indicating that the microstrain also has a complex heterogeneity.

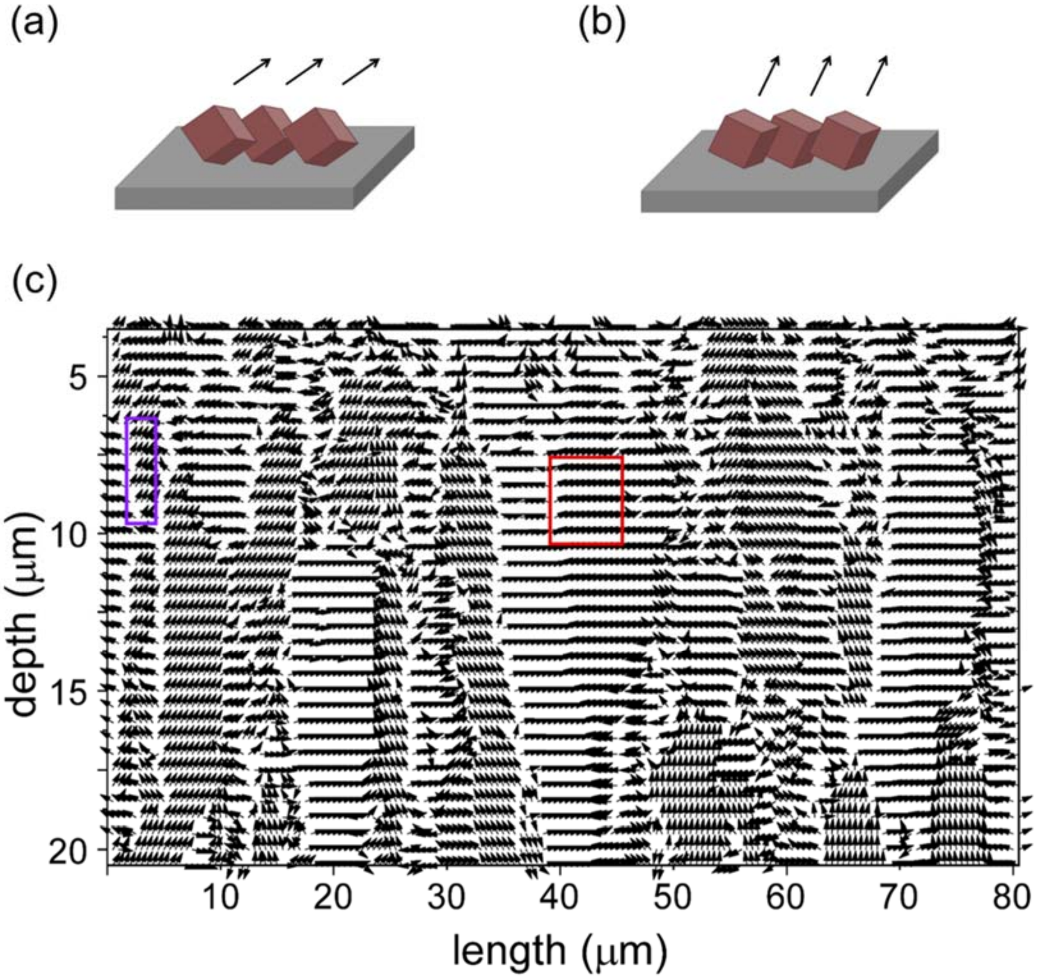

Supplementary Figure 19. Two dominant crystal orientations of the (100) perovskite plane. Sketch illustration for the two dominant orientations of (a) 35° and (b) 64°. Red and purple boxes in (c) indicate preferred orientations of the (100) perovskite plane at around 35° and 64°. In the cubic structure, the (111) plane intersects the (100) plane at 54.7°, which leads to the conclusion that the (111) plane is oriented normal to the substrate. Similarly, the (210) plane intersects the (100) plane at 26.57°, thereby the (210) plane being oriented normal to the substrate.

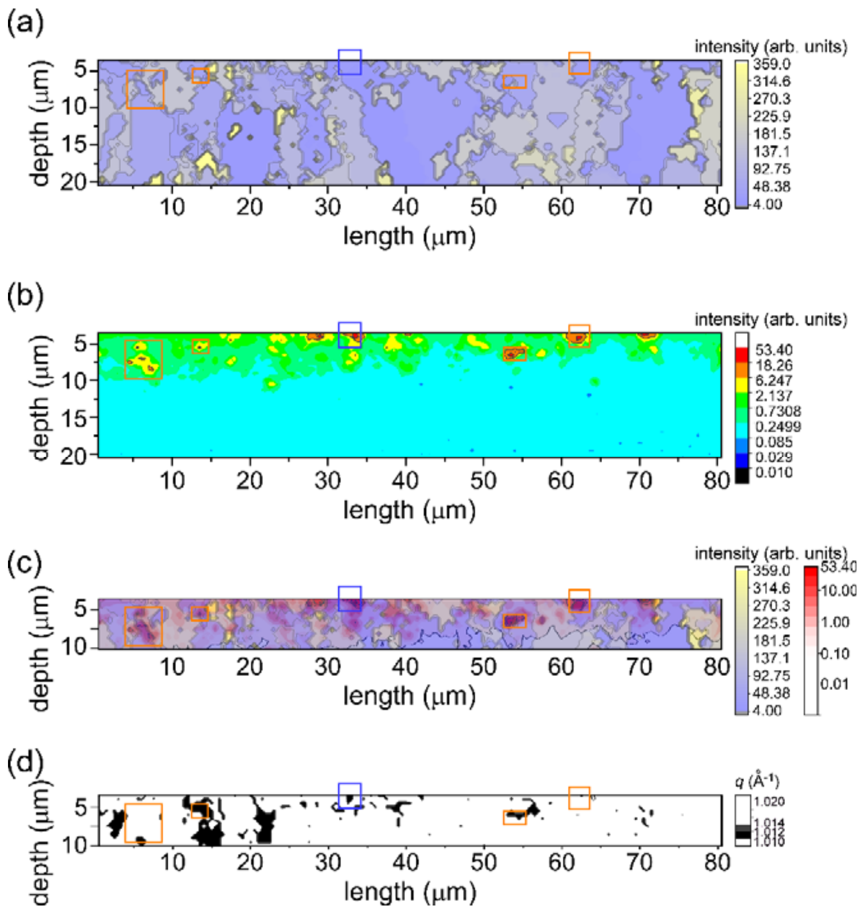

Supplementary Figure 20. Correlations between the moisture degradation, grain structures and the strain concentration. 80 × 20 μm^2 (length × depth) intensity maps of (a) the preferred orientation of the (100) perovskite peak and (b) the PbI_2 (100) peak, (c) overlap between the intensity maps of the preferred orientation of the (100) perovskite peak and the PbI_2 (100) peak, and (d) local *q* position variations of the (100) perovskite peak reflecting the strain concentration. The depth and length are in the same scale bar. Overall, longitudinal shapes are observed in (a), indicating that the major perovskite grains grow along the vertical direction. The blue box denotes the degradation region within a single grain at the surface, and the orange boxes denote the degradation at the grain boundary. According to the strain estimation mentioned above, the lower *q* position reflects strain

with a higher magnitude. We correlate the lower local *q* positions of the (100) perovskite peak (d) with the intensity map of the PbI_2 (100) peak (b). We note that the high degradation-intensity (PbI_2) regions are related to the local strain centration at a certain degree. Hence, these correlations indicate that the strain can accelerate the degradation, and importantly, it may provide the decomposition starting point at grain boundaries or surface.

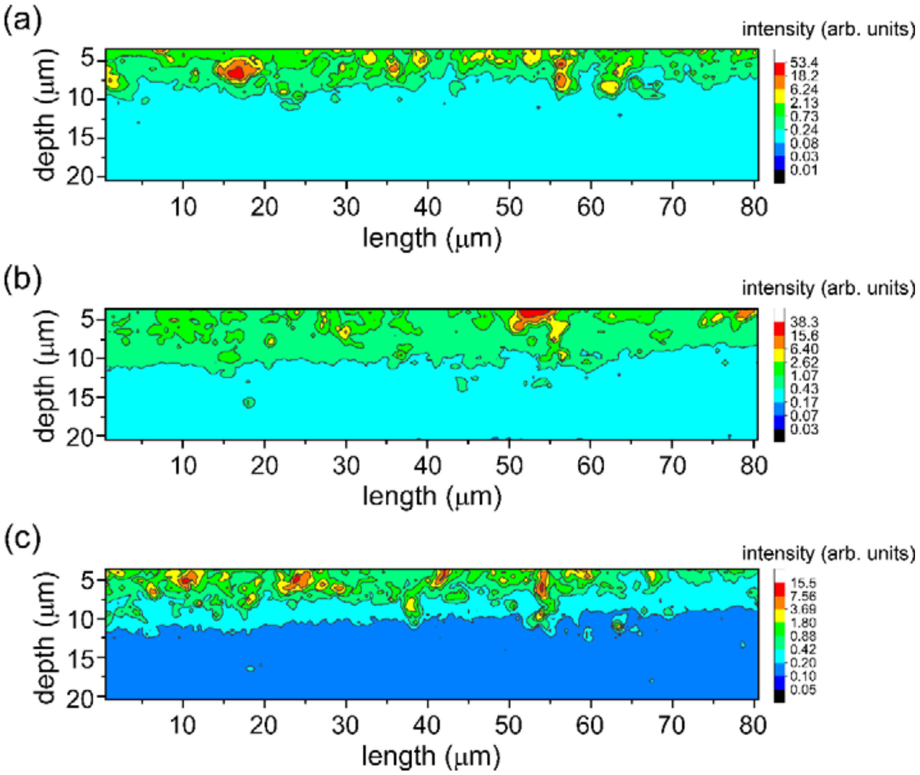

Supplementary Figure 21. 80 × 20 μm^2 (length × depth) intensity maps of degradation products. (a) monohydrate, (b) di-hydrate, and (c) $CH_3NH_3Br/CH\ (NH_3)_2I$.

Supplementary References

1. Williamson, G. K. & Hall, W. H. X-ray line broadening from filed aluminium and wolfram. *Acta Metall.* **1,** 22–31 (1953).

2. Jones, T. W. *et al.* Lattice strain causes non-radiative losses in halide perovskites. *Energy Environ. Sci.* **12,** 596–606 (2019).

3. Xue, D.-J. *et al.* Regulating strain in perovskite thin films through charge-transport layers. *Nat. Commun.* **11,** 1514 (2020).

4. Feng, J. Mechanical properties of hybrid organic-inorganic $CH_3NH_3BX_3$ (B = Sn, Pb; X = Br, I) perovskites for solar cell absorbers. *APL Mater.* **2,** 81801 (2014).

5. Liu, D. *et al.* Strain analysis and engineering in halide perovskite photovoltaics. *Nature Materials* **20,** 1337–1346 (2021).

6. Pratap, S., Schlipf, J., Bießmann, L. & Müller-Buschbaum, P. Hierarchical structures from nanocrystalline colloidal precursors within hybrid perovskite thin films: Implications for photovoltaics. *ACS Appl. Nano Mater.* **3,** 11701–11708 (2020).

7. Maughan, A. E. *et al.* Anharmonicity and octahedral tilting in hybrid vacancy-ordered double perovskites. *Chem. Mater.* **30,** 472–483 (2018).

8. Hao, F., Stoumpos, C. C., Liu, Z., Chang, R. P. H. & Kanatzidis, M. G. Controllable perovskite crystallization at a gas-solid interface for hole conductor-free solar cells with steady power conversion efficiency over 10%. *J. Am. Chem. Soc.* **136,** 16411–16419 (2014).

9. Leguy, A. M. A. *et al.* Reversible hydration of $CH_3NH_3PbI_3$ in films, single crystals, and solar cells. *Chem. Mater.* **27,** 3397–3407 (2015).

10. Xie, L. Q. *et al.* Understanding the cubic phase stabilization and crystallization kinetics in mixed cations and halides perovskite single crystals. *J. Am. Chem. Soc.* **139,** 3320–3323 (2017).

11. Vincent, B. R., Robertson, K. N., Cameron, T. S. & Knop, O. Alkylammonium lead halides. part 1. isolated PbI_6^{4-} ions in $(CH_3NH_3)_4PbI_6.2H_2O$. *Can. J. Chem.* **65,**

1042–1046 (1987).

12. Imler, G. H. *et al.* Solid state transformation of the crystalline monohydrate $(CH_3NH_3)PbI_3(H_2O)$ to the $(CH_3NH_3)PbI_3$ perovskite. *Chem. Commun.* **51,** 11290–11292 (2015).

13. Prathapani, S., Choudhary, D., Mallick, S., Bhargava, P. & Yella, A. Experimental evaluation of room temperature crystallization and phase evolution of hybrid perovskite materials. *CrystEngComm* **19,** 3834–3843 (2017).

14. Noh, J. H., Im, S. H., Heo, J. H., Mandal, T. N. & Seok, S. I. Chemical management for colorful, efficient, and stable inorganic-organic hybrid nanostructured solar cells. *Nano Lett.* **13,** 1764–1769 (2013).

15. Pistor, P., Burwig, T., Brzuska, C., Weber, B. & Fränzel, W. Thermal stability and miscibility of co-evaporated methyl ammonium lead halide ($MAPbX_3$, X = I, Br, Cl) thin films analysed by: In situ X-ray diffraction. *J. Mater. Chem. A* **6,** 11496–11506 (2018).
