# Supplementary material for: Mapping structure heterogeneities and visualizing moisture degradation of perovskite films with nano-focus WAXS
Source: Nat Commun. 2022 Nov 5;13:6701. doi: 10.1038/s41467-022-34426-y (PMC9637205; doi:10.1038/s41467-022-34426-y)
Supplement: Supplementary file 3 — Description of Additional Supplementary Files [file 41467_2022_34426_MOESM3_ESM.pdf]

File name: Supplementary Data 1

Description: CIF files either from the published reference or Crystallography Open Database/Cambridge Crystallographic Data Centre to simulate XRD patterns.
